# Molecular characterization of *FAM222B* as a novel disease gene for dominant cardiovascular laterality defects

**DOI:** 10.1038/s41598-026-64853-6

**Published:** 2026-08-22

**Authors:** Nina Reitz, Jessica Lambertz, Öznur Yilmaz, Tobias T. Lindenberg, Khadija Channab, Birgit Rau, Stefanie Ramrath, Enrico Mingardo, Hanna Schöpper, Bettina C. Kirchmaier, Matthias Geyer, Gabriel C. Dworschak, Johannes Breuer, Nicole Müller, Birthe Schaidinger, Alina C. Hilger, Julia Hoefele, Korbinian M. Riedhammer, Marc-Phillip Hitz, Gregor Dombrowsky, Hashim Abdul-Khaliq, Ulrike M. M. Bauer, Lars Fester, Heiko M. Reutter, Katinka Breuer, Benjamin Odermatt

**Affiliations:** 1 Institute of Anatomy, Neuroanatomy, Faculty of Medicine, University of Bonn, 53115 Bonn, Germany; 2 Department of Otorhinolaryngology, University Medical Center Bonn (UKB), 53127 Bonn, Germany; 3 Institute of Anatomy, Anatomy and Cell Biology, Faculty of Medicine, University of Bonn, 53115 Bonn, Germany; 4 Faculty of Physics, Mathematics and Computer Science, Applied Computer Science, University of Bayreuth, 95447 Bayreuth, Germany; 5 Faculty of Life Science: Food, Nutrition & Health, Department of Nutritional Biochemistry, University of Bayreuth, 95447 Bayreuth, Germany; 6 Buchmann Institute for Molecular Life Sciences (BMLS), Institute of Cell Biology and Neuroscience, Goethe University Frankfurt, 60438 Frankfurt Am Main, Germany; 7 Institute of Structural Biology, Faculty of Medicine, University of Bonn, 53127 Bonn, Germany; 8 Department of Neuropaediatrics, University Hospital Bonn, 53127 Bonn, Germany; 9 Division of Paediatric Cardiology, Department of Paediatrics and Adolescent Medicine, University Hospital Bonn, 53127 Bonn, Germany; 10 Department of Paediatrics and Adolescent Medicine, Friedrich-Alexander University of Erlangen-Nuremberg, 91054 Erlangen, Germany; 11 Institute of Human Genetics, LMU University Hospital, LMU Medizin, Ludwig-Maximilians- Universität München, 80336 Munich, Germany; 12 Institute of Human Genetics, Klinikum Rechts Der Isar, Technical University of Munich, TUM School of Medicine and Health, 81675 Munich, Germany; 13 Division of Nephrology, Department of Paediatrics, Boston Children’s Hospital, Harvard Medical School, Boston, MA USA; 14 Center for Rare and Genetic Kidney Diseases, Department of Nephrology, TUM University Hospital Rechts Der Isar, TUM School of Medicine and Health, 81675 Munich, Germany; 15 Institute for Medical Genetics, Carl-Von-Ossietzky-University, 26133 Oldenburg, Germany; 16 Institute of Paediatric Cardiology, Department of Paediatrics and Adolescent Medicine, Saarland University Hospital/ Medical Center, 66421 Homburg/ Saar, Germany; 17 Competence Network for Congenital Heart Defects, 13353 Berlin, Germany; 18 National Register for Congenital Heart Defects, 13353 Berlin, Germany; 19 Institute of Neonatology and Paediatric Intensive Care, Department of Paediatric and Adolescent Medicine, Friedrich-Alexander University of Erlangen-Nürnberg, 91054 Erlangen, Germany; 20 Department of Neonatology and Paediatric Intensive Care, Altonaer Children’s Hospital, 22763 Hamburg, Germany

**Keywords:** Cardiogenesis, Zebrafish, *fam222ba*, *fam222bb*, Heart looping, Nemo-like kinase, Cardiology, Diseases, Genetics, Medical research

## Abstract

Cardiovascular laterality defects occur with an estimated birth prevalence of 1.1/10,000 live births, associated with congenital heart defects (CHD) and situs abnormalities. Known disease genes explain about 20% of all cases often correlated with primary ciliary dyskinesia (PCD). We aimed to identify disease genes beyond PCD-related aetiologies using exome sequencing in 16 case-parent trios followed by exome survey in 2,109 individuals with situs inversus totalis, heterotaxy, or isolated CHD. We identified six different variants in *FAM222B*, previously discussed as a candidate gene for cerebral cavernous malformations*.* The variant c.899G > A (p.300Arg > His) was found de novo in two unrelated families. FAM222B has been described as a substrate of Nemo-like kinase (NLK), associated with left–right body axis determination. Structural modelling suggests a function of FAM222B Arg300 in NLK recognition. We investigated whole-mount in situ hybridization (WISH) expression pattern and genomic context of the zebrafish (zf) *FAM222B* homologues, *fam222ba/bb,* and *fam222aa*. Using a double-knockout (dd-KO) *fam222ba/bb* zf line, we identified aberrant cardiac looping in these larvae and enlarged atrium and ventricle in adult zf. We further tested the c.899G > A variant using human mRNA injections in *Tg(kdrl:EGFP)* wildtype (wt) reporter-zf, which led to perturbed cardiogenesis. Together, we propose *FAM222B* as a novel candidate gene for cardiovascular laterality defects.

**Supplementary Information:**

The online version contains supplementary material available at 10.1038/s41598-026-64853-6.

## Introduction

Cardiovascular laterality defects are rare congenital anomalies of embryonic left–right axis patterning with a reported birth prevalence of about 1.1 in 10,000 live births^1^. Despite decades of progress in both pre- and postnatal diagnostic approaches and surgical treatment of cardiovascular laterality defects, the condition remains challenging for clinicians^2^. While individuals with situs inversus totalis do not experience physical impairments despite completely mirrored visceral organs, individuals with heterotaxy, particularly situs ambiguous, are in 90% associated with complex CHD, which remain challenging in terms of surgical reconstruction^3^. The affected individuals may also present with pulmonary situs abnormalities (bilateral bi-lobed lungs) or abdominal situs abnormalities (e.g. mal-positioned liver, poly- or asplenia). Besides complex cardiovascular laterality defects, estimates suggest that about 3–7% of all isolated congenital heart defects (CHD) are due to abnormal embryonic left–right axis patterning comprising double outlet right ventricle (DORV), atrioventricular septal defect (AVSD) or transposition of the great arteries (TGA)^4^.

Determination of left–right body axis during embryogenesis commences at the early somite state. To this point the embryonic development is completely symmetric. The break in this symmetric development is primarily steered by a leftward flow generated by rotating primary cilia of the primitive node. Nodal cilia play a major role in forming asymmetry and organogenesis of unpaired organs as heart, liver or spleen generating a leftward flow of extracellular fluid, also called nodal flow^5,6^. Through clockwise rotation, nodal cilia produce a leftward nodal flow, which triggers a downstream signal cascade in the lateral plate mesoderm. Disruption of genetic drivers that govern determination of left–right body axis during embryogenesis may cause complex laterality defects or isolated CHD only^7^. The known genetic background of laterality defects explains about 20% of cases and comprises (de novo) dominant monoallelic, recessive biallelic, and X-linked variants^4,8,9^. So far, primary ciliary dyskinesia (PCD) represents a main underlying cause of laterality defects^10^ .

In order to identify new disease genes for cardiovascular laterality defects without PCD background, we analysed the exomes of 16 case-parent trios with cardiovascular laterality defects in which PCD was excluded prior to exome analysis. Here, we previously identified and described two novel variants in laterality defects associated disease genes *PKD1L1* and *ZIC3* as well as ultra-rare biallelic variants in *LMBRD1* and *DNAH17* and one ultra-rare de novo variant in *WDR47* suggesting all three as novel candidate genes^11^. In two case-parent trios we found an identical de novo ultra-rare missense variant (dbSNP rs753661690, c.899G > A, p.Arg300His) in the *FAM222B* gene.

Single exome survey of 2,109 individuals with situs inversus totalis, heterotaxy, or isolated CHD identified five additional variants in *FAM222B* in five independent families, including one novel variant. *FAM222B* is localised on chromosome 17 and encodes a ~ 60 kDa nucleoplasmic and mitochondrial protein of yet unknown function (UniProt database). Computational analysis of the predicted protein sequence so far did not reveal any typical signal-peptide, transmembrane nor membrane-anchor regions^12^. *FAM222B* has been discussed as a candidate gene for cerebral cavernous malformations before^12^, still its protein function and potential role in embryonic development remains elusive and has not been described in the context of CHD.

To characterize the role of *FAM222B* on cardiac development, we examined its expression, function and genetic context in the zebrafish (zf) model. In previous analyses, expression of both zf *fam222b* paralogues and *fam222aa* could be confirmed in the cardiac region of the zebrafish larvae (zfl), especially at 48 h post fertilization (hpf), the time period of cardiac looping (daniocell). In single cell transcriptional analysis of mice embryos between 9.5 and 13.5 days of gestation, expression of *Fam222b* could clearly be detected in cardiac muscle lineages (Mouse Organogenesis Cell Atlas (MOCA)). To gain further insights into the effect of zf *fam222b* paralogues on cardiogenesis, we further examined heart looping in zfl as well as cardiac morphology and histology in adult zf’ hearts using a TALEN-generated double-knockout (dd-KO) zf line for *fam222ba/bb*. Here, we focused on the de novo variant, *FAM222B* SNV: 17–28,759,060-G-A; GRCh38, dbSNP rs753661690, c.899G > A, p.300Arg > His, which occurred de novo in two unrelated families and is in close spatial proximity to the FAM222B phosphorylation-site Ser296. Using structural modelling we find that p.300Arg is predicted to be involved in an interaction with the β-sandwich region of the N-lobe of Nemo-like kinase (NLK). NLK though has been associated to human embryonic heart development and left–right axis determination before. It has been shown that NLK regulates the Wnt signalling pathway^13^ which plays a role in several steps of left–right determination in development^14^. Furthermore, Nlk is proposed to be an essential co-activator of Wnt signalling during early zf development, too^15^. It is also involved in human phosphorylation and ensuing inhibition of CREB binding protein (CBP)^16^ and NOTCH^17^ which both as well take part in cardiac development (CBP^18^; NOTCH^19^). In order to advance the understanding of a possible pathogenic effect of the missense variant c.899G > A in our affected patients, we performed poly(A) mRNA injections for overexpression in fluorescent wildtype (wt) reporter-zf embryos. Our results suggest *FAM222B* to be involved in cardiac development and in human cardiovascular laterality defects.

## Results

### Exome analysis of case-parent trios with cardiovascular laterality defects

**Fig. 1:**
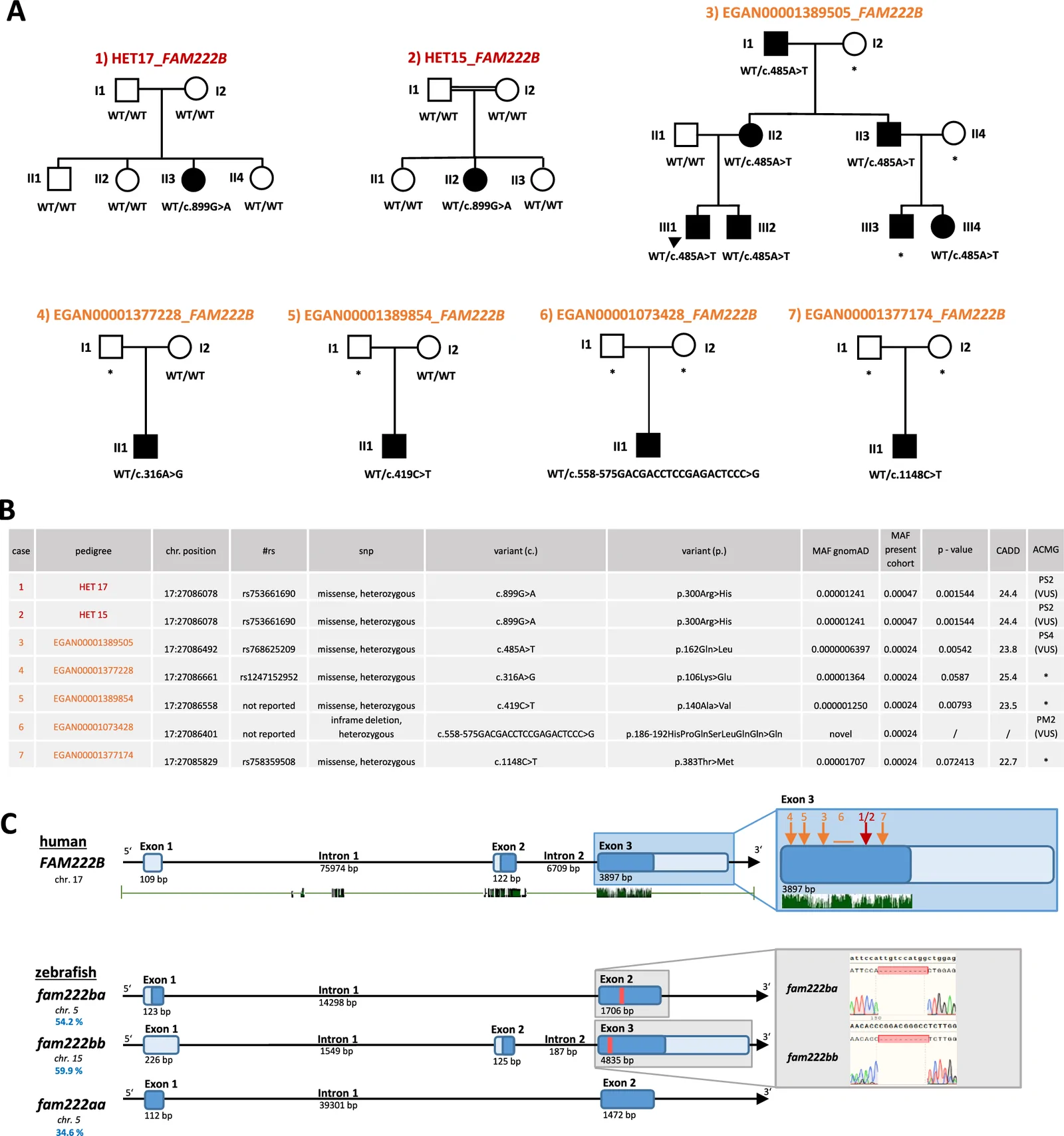
Patient pedigrees and genomic comparison of zf and human FAM222B. (**A**) Pedigrees of cases with cardiovascular laterality defects, situs inversus totalis, heterotaxy, or isolated CHD identified through exome sequencing. Circle for female, square for male. Doubled line for consanguineous marriage. Affected patients in black: I1: lifelong reduced physical capacity, late diagnosis of an unspecified ASD; II2: PDA, patent foramen ovale (PFO); II3: congenital valvular pulmonary stenosis; III1: VSD, PDA, ASD II, bicuspid aortic valve with stenosis and insufficiency; III2: multiple VSDs, double chambered right ventricle, PDA, bicuspid aortic valve with insufficiency; III3: VSD, PDA, PFO; III4: common truncus arteriosus type A1, ASD II, left superior vena cava. * = No DNA available (**B**) Variant table of FAM222B including specific changes in base and amino acid sequence, MAF, CADD and ACMG, VUS = variant of uncertain significance, * cannot be classified according to ACMG criteria. (C) Genetic structure of human *FAM222B*, the zf homologues *fam222ba/bb* and zf *fam222aa.* The introns (lines), exons (boxes) and protein coding (dark blue) sequence sizes were adjusted according to the entire gene. Dark green bands underneath the human *FAM222B* reveal the genetic conservation score compared to zf (UCSC genome browser). The six variants identified in our studies are marked with an arrow (point-mutations) or line (deletion) in the highly conserved exon three. Variant 1/2, which was further observed in our studies, was highlighted in red; other variants were highlighted in orange. Red bars in the zf paralogues reveal the position of the 10 bp deletion in the TALEN-generated *fam222ba/bb* dd-KO line, leading to a reading frame shift with a resulting early stop codon. Confirmative sequencing of the received dd-KO zfl is shown in the box aside. Blue numbers beneath each chromosome label represent a comparative analysis between human FAM222B and zf homologues Fam222ba, Fam222bb as well as zf Fam222aa on amino acid level using the UniProt align tool. The highest similarity was revealed between FAM222B and Fam222bb (59.9%), followed by Fam222ba (54.2%) and Fam222aa (34.6%).

As outlined above, the results of exome sequencing of 14 case-parent trios were already published by Breuer et al. in 2022^11^. In two further case-parent trios (HET15 and HET17) we found an identical de novo ultra-rare missense variant in the *FAM222B* gene (Fig. 1A, cases 1&2). This variant has been identified applying the same filter criteria as previously described by Breuer et al.^11^. The *FAM222B* variant SNV: 17–28,759,060-G-A (GRCh38, dbSNP rs753661690, c.899G > A, p.Arg300His) has been reported in only 20 of 1.612.112 alleles (minor allele frequency, MAF 0.00001241) without reports of homozygotes (gnomAD v4.1.0). The CADD score is 24.4. Both affected individuals presented with left isomerism and complex CHD. Screening of homozygous variants in HET15 with consanguineous marriage did not yield any hits.

### Exome survey of 2,109 single exomes in individuals with situs inversus totalis, heterotaxy, or isolated CHD

Using the same filter criteria as applied for our unbiased exome survey in case-parent trios, we surveyed 2,109 single exomes of individuals with situs inversus totalis, heterotaxy, or isolated CHD and identified five additional variants in *FAM222B* in five independent families (Fig. 1A, cases 3–7; Fig. 1B). The five variants comprised four heterozygous missense variants and one in-frame deletion of seven amino acids followed by one amino acid insertion. The affected variant carriers presented with isolated heart defects such as atrial septal defect (ASD), ventricular septal defect (VSD), atrioventricular septal defect (AVSD) and bicuspid aortic valve. More detailed filtering of exome data in these five families did not identify another plausible variant in previously established disease genes associated with congenital heart defects.

In individual “III-1, EGAN00001389505”, who presented with congenital VSD, patent ductus arteriosus (PDA), ASD II and bicuspid aortic valve with stenosis and insufficiency, we found a missense variant c.485A > T (p.Gln162Leu, NM_001077498.3, rs768625209) with a CADD score of 23.8 (Fig. 1A, case 3). The variant has been reported only once in 1.563.132 alleles (MAF 0.0000006397) without reports of homozygotes (gnomAD v4.1.0). Within a three-generation multiplex family, the variant segregated between all seven differently affected family members. All PDAs occurring in this family were treated with interventional catheter or were surgically ligated in early infancy.

In individual “EGAN00001377228”, who presented with congenital ASD, we found a missense variant c.316A > G (p.106Lys > Glu, NM_001077498.3, rs1247152952) with a CADD score of 25.4 (Fig. 1A, case 4). The variant has been reported in 22 of 1.613.164 alleles (MAF 0.00001364) without reports of homozygotes (gnomAD v4.1.0). Genetic material of the father was not available.

In individual “EGAN00001389854”, who presented with congenital bicuspid aortic valve, we found a missense variant c.419C > T (p.140Ala > Val, NM_001077498.3) with a CADD score of 23.5 (Fig. 1A, case 5). The variant has been reported in only 2 of 1.600.126 alleles (MAF 0.000001250) without reports of homozygotes (gnomAD v4.1.0). Genetic material of the father was not available.

In individual “EGAN00001073428”, who presented with congenital AVSD, we found an in-frame deletion of seven amino acids followed by one amino acid insertion c.558_575delGACGACCTCCGAGACTCCC > G (p.186_192delHisProGlnSerLeuGlnGln > Gln, NM_001077498.3) in a conserved region of FAM222B (Fig. 1A, case 6). This deletion has not been previously described. Parental DNA was not available.

In individual EGAN00001377174, who presented with congenital ASD, we found a missense variant c.1148C > T (p.383Thr > Met, NM_001077498.3, rs758359508) with a CADD score of 22.7 (Fig. 1A, case 7). The variant has been reported in 27 of 1.581.696 alleles (MAF 0.00001707) without reports of homozygotes (gnomAD v4.1.0). Parental DNA was not available.

All missense variants reported in our study have been previously reported in gnomAD with a MAF ranging from < 0.00001707 to 0.0000006397 compared to a MAF of 0.000235 for all missense variants in families 3, 4, 5 and 7 or 0.000470 for families 1 and 2 in the present sample here (combined cohort of discovery sample and single exomes). The frequencies of the missense variants identified in families 1, 2, 3, and 5 were significantly higher in the present case cohort than in the general population. In contrast, in families 4 and 7, the identified variant occurred at a comparable frequency in the combined cohort and the general population. The nonsense indel variant identified in family 6 is not represented in the gnomAD at all (Fig. 1B).

Using the UCSC genome browser we found all of our patients ‘ *FAM222B* variants localized in the highly conserved exon 3 region (Fig. 1C).

### Functional analysis of *FAM222B* in the zebrafish larvae model

For a better understanding of our candidate gene function, we chose the zfl *(danio rerio)* as an in vivo development model. To do so, we analysed the gene structure of human *FAM222B* and compared it to the zf *fam222b* homologues and *fam222aa* (Fig. 1C). We further reviewed the conservation of human FAM222B compared to zf using the UniProt align tool. The highest similarity on amino acid level between human FAM222B and zf was observed with 59.9% for Fam222bb. This was followed by Fam222ba with 54.2% and Fam222aa with 34.6% (Fig. 1C). Comparative analysis of the amino acid conservation of the observed human variant gene-loci in zf revealed the highest conservation in zf Fam222bb with three out of six human variant loci preserved here (see Supplementary S1 online). Notably, the gene-locus of our human variant of interest 1/2, rs753661690, p.300Arg > His is preserved in zf Fam222bb as well as in zf Fam222ba, prompting this variant as best suitable for our further analysis. Genomic comparison of up- and downstream neighbouring genes of human *FAM222B*, with the zf’ homologues *fam222ba/bb* and *fam222aa* shows the highest conservation in this case with *fam222ba* followed by *fam222bb* and no common gene neighbours with *fam222aa* (see Supplementary S2 online).

Control sequencing of the obtained TALEN-derived *Tg(kdrl:eGFP; gata ds:red) fam222bb* hu10539; *fam222ba* hu10755; s843Tg (ZDB-FISH-161130–1) dd-KO strain is concordant to the given 10 bp deletion from our collaborators and zfin (Fig. 1C).

### Whole-mount in situ hybridization (WISH) of *fam222ba/bb* in zf cardiac development

**Fig. 2:**
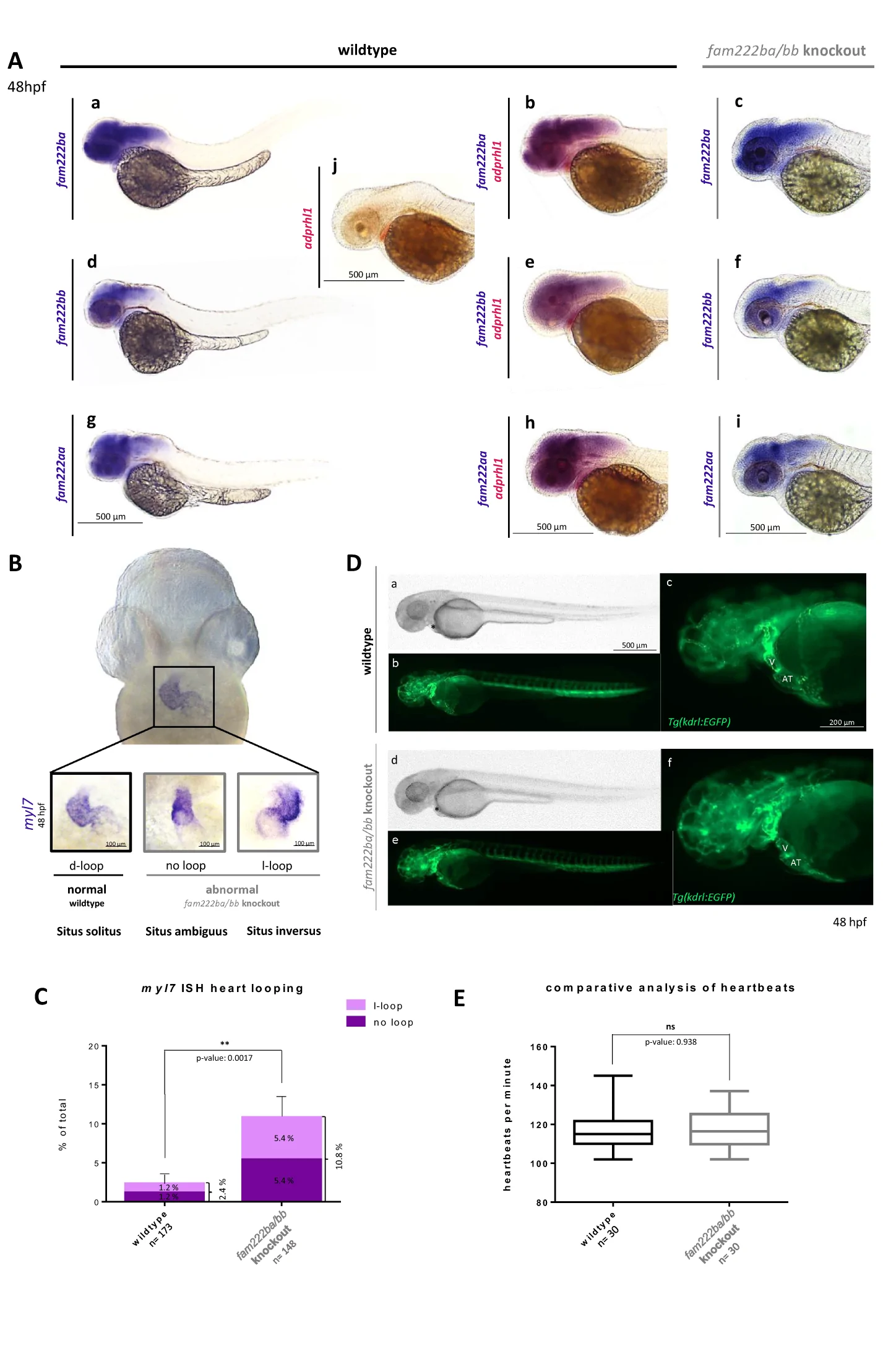
Whole-mount in situ hybridization (WISH) analysis against *fam222* homologues and cardiac markers *adprhl1* and *myl7* in wt and *fam222ba/bb* dd-KO zfl at 48 hpf. (**A**) Expression of *fam222* homologues at 48 hpf using three different probes against each zf *fam222* gene*: fam222ba* (a-c), *fam222bb* (d-f), *fam222aa* (g-i). Staining in wt zfl (a,d,g) compared to *fam222ba/bb* dd-KO zfl (c,f,i). Blue WISH staining is apparent in the zf head region as well as in the pericardium region including the heart. Additional red staining of the heart was performed using a probe against cardiac *adprhl1* (j). Double staining was realized in wt zfl (b,e,h) in order to test for overlay of *fam222* in blue and *adprhl1* in red. Scale bar at the bottom applies for whole row above: 500 µm. (**B**) WISH-analysis to examine heart laterality defects. *Myl7* indicates cardiac looping in wt and *fam222ba/bb* dd-KO zfl at 48 hpf. Analysis of various ways of cardiac looping: d-loop (right, normal), no loop (mid), l-loop (left, inversed). Scale bar 100 µm. (**C**) Direction and amount of false directed heart looping in wt and *fam222ba/bb* dd-KO zfl (N = 6, n = 148–173 in each group, in total), no loop in purple, l-loop in lavender. Two-sided Chi^2^ test: p-value: 0.0017, ** = very significant. (**D**) Comparative analysis of whole-mount *Tg(kdrl:EGFP)* wt and *Tg(kdrl:EGFP) fam222ba/bb* dd-KO zfl at 48 hpf showed no difference in fluorescent in vivo imaging concerning morphological and cardiovascular development between both groups. Lateral view of whole-mount zfl in bright field (a,d). (b,e) Lateral view of whole-mount zfl in fluorescence mode (b,e). Lateral view of zoomed in cranial head & heart region of whole-mount zfl in fluorescence mode (c,f). Abbreviations: asterisks* = heart, AT = atrium, V = ventricle. Scale bar for (a,b,d,e): 500 µm. Scale bar for (c,f): 200 µm. (**E**) Comparison of heartbeats per minute reveals no difference between wt and dd-KO zfl (N = 3, n = 30 in each group). Median (bpm): wt: 115, dd-KO: 116.5. Unpaired two-tailed t-test: p-value: 0.938, ns = not significant.

For analysis of *fam222ba*, *fam222bb* and *fam222aa* mRNA expression patterns in wt and *fam222ba/bb* dd-KO zfl, we performed whole-mount in situ hybridization throughout various time-points between 12 and 72 hpf (see Supplementary S3 online). Due to highest overall expression patterns in 48 hpf old zfl, further investigations were performed at this time-point (Fig. 2A). Expression patterns of all three *fam222* homologues were restricted to the head and the cardiac region in a similar pattern (Fig. 2A). There were no obvious expression differences in any of the *fam222* homologue stainings for the *fam222ba/bb* dd-KO (Fig. 2A: c,f,i) compared to wt zfl (Fig. 2A: a,d,g). To closer proof expression of *fam222* especially in the developing zfl heart, double staining against ADP-Ribosylhydrolase Like 1 enzyme (*adprhl1*) as a cardiac marker was performed in wt, displaying a clear overlay of both probes in the cardiac region, supporting the role of *fam222ba/bb* as a regulator of cardiac development (Fig. 2A: b,e,h). WISH analysis at different time-points revealed additional staining in the cloaca region for *fam222ba* at 24 hpf and in the pineal gland for *fam222bb* at 72 hpf (see Supplementary S3 online).

To further investigate the role of zf *fam222ba/bb* in heart development and looping, we performed a WISH-analysis at 48 hpf comparing wt and *fam222ba/bb* dd-KO zfl using an anti-*myl7* probe to detect heart laterality defects (Fig. 2B). Our data strongly showed higher frequency of cardiac looping defects in dd-KO zf hearts compared to wt zfl. Whereas heart looping in wt mostly presented itself as normally d-looped (97.6%), the amount of properly d-looped hearts was significantly lower in *fam222ba/bb* dd-KO zfl (89.2%), p-value: < 0.01**. In both groups, wt and dd-KO, the distribution of l-looped and no looped hearts was equal (Fig. 2C). Based on these results, our data strongly supports *fam222ba/bb* dd-KO in modulating heart looping.

Comparative in vivo imaging analysis focusing on the morphological and cardiovascular development between whole-mount *Tg(kdrl:EGFP)* wt and *Tg(kdrl:EGFP) fam222ba/bb* dd-KO zfl at 48 hpf revealed no obvious difference between both groups (Fig. 2D). In addition to this, also the heart rate (in beats per minute, bpm, at 48 hpf of the zfl) showed no significant difference between the two groups wt and dd-KO (p-value: > 0.05, Fig. 2E). Moreover, no cardiac arrhythmias were observed in any group.

### Cardiac analysis of *fam222ba/bb* knockout in adult zebrafish

**Fig. 3:**
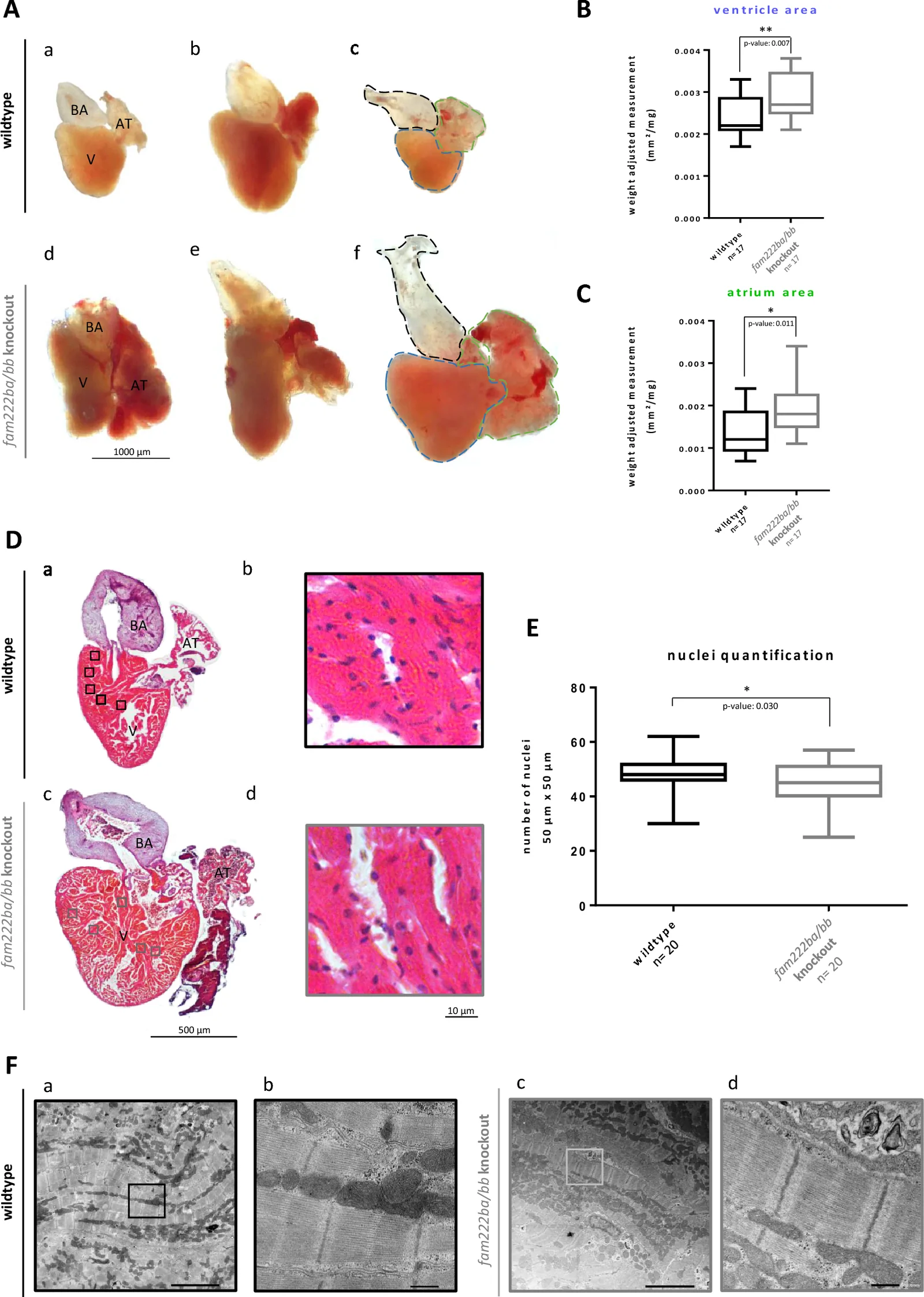
Examination of adult zf hearts, morphology and histology. (**A**) Detailed examination and comparison of dissected adult wt and *fam222ba/bb* dd-KO zf ‘ hearts (N = 4, n = 17 in each group, except for BA analysis in dd-KO zf group: N = 4, n = 16, in total): (a-c) show representative examples of normally developed wt zf hearts, (d-f) demonstrate various representative altered *fam222ba/bb* dd-KO zf hearts. (d) presents a hypertrophic atrium, (e) shows a slim ventricle and (f) reveals a hypertrophic BA. Scale bar: 1000 µm. Dashed lines symbolize the way of measurement exemplary in c&f for the zf ‘ ventricle in blue, the atrium in green and the BA in black. Abbreviations: AT = atrium, BA = bulbus arteriosus, V = ventricle. No significant difference between BA area of wt and *fam222ba/bb* dd-KO zf. Median (mm^2^/ mg): wt: 0.0010, dd-KO: 0.0011. Unpaired two-tailed t-test: p-value: 0.366, ns = not significant. (**B**) Box plot graph comparing wt and *fam222ba/bb* dd-KO zf ‘ ventricle area. Median (mm^2^/ mg): wt: 0.0022, dd-KO: 0.0027. Unpaired two-tailed t-test: p-value: 0.007, ** = very significant. (**C**) Box plot graph comparing wt and *fam222ba/bb* dd-KO zf ‘ atrium area. Median (mm^2^/ mg): wt: 0.0012, dd-KO: 0.0018. Unpaired two-tailed t-test: p-value: 0.011, * = significant. (**D**) HE-Staining of adult wt (a,b) and dd-KO (c,d) zf hearts. Each box in the overview (a,c) corresponds to a 50 µm × 50 µm area used for quantification of nuclei. Both areas enclosed by thicker (bold) boxes are presented examples at higher resolution in the corresponding magnified panel (b,d). Scale bar: overview: 500 µm, zoom: 10 µm. Abbreviations: AT = atrium, BA = bulbus arteriosus, V = ventricle. (**E**) Box plot graph comparing the number of nuclei in each selected wt and *fam222ba/bb* dd-KO heart in five 50 µm × 50 µm areas of ventricular myocardium (N = 4, n = 20). Median: wt: 48, dd-KO: 45. Unpaired two-tailed t-test: p-value: 0.030, * = significant. (**F**) TEM images showing ventricular myocardium of adult wt (a,b) and *fam222ba/bb* dd-KO (c,d) zf. Overview (a,c) and higher magnification (b,d) of sarcomeres. Box in overview indicates zoomed section. There was no apparent difference neither in the overall tissue assembly nor in the detailed composition of sarcomeres. Scale bar: overview: 5 µm, zoom: 500 nm.

Detailed examination and comparison of adult (≈ two-year-old) wt and *fam222ba/bb* dd-KO zf hearts showed structural anomalies in the anatomical composition and proportion of the dd-KO zf hearts. For analysis of proportion, area measurements were adjusted to individual zf weights. There was no significant difference between wt and *fam222ba/bb* dd-KO weights, p-value: > 0.05 (see Supplementary S4 online). In some dd-KO zf, the bulbus arteriosus (BA) seemed enlarged (Fig. 3A f), however, this phenotype did not appear in a significant difference in comparison to wt zf (p-value: > 0.05). One out of 16 analysed dd-KO zf presented itself with a doubled BA, no anomalies were observed in total 17 wt zf BA (see Supplementary S5 online).

Remarkably, adult dd-KO zf presented with enlarged hypertrophic ventricle and atrium area compared to adult wt zf in our 2D measurements (Fig. 3A,B, p-value: < 0.01**; Fig. 3A,C, p-value: < 0.05*).

Unexpectedly the amount of cell nuclei in adult dd-KO ventricular heart tissue was significantly decreased (Fig. 3D,E, p-value: < 0.05*). Cell density and tissue area were quantified in dd-KO and WT zf heart samples. Notably, for both groups analysis areas with approximately confluent cellular organization without detectable intercellular spaces were selected. The reduced cell density observed in dd-KO heart tissue compared to WT (44.63 vs. 47.95 cells per 2500 µm^2^) strongly suggests an increase in average cell size. This is consistent with a hypertrophic phenotype at the cellular level. In addition to that, the total tissue area normalized to organismal mass was higher in KO than in WT (0.002929 vs. 0.002422 mm^2^/mg). By integrating these metrics, the estimated number of cells per mg was calculated in our studies, revealing a higher overall cell number in KO compared to WT (0.1307 vs. 0.1161 cells/mg), corresponding to an increase of approximately 12.6%, which indicates cellular hyperplasia in dd-KO zf tissue.

Through Masson’s Trichrome Stain it was also ensured that no sample was biased by fibrous tissue infiltration. With the exception of the physiological fibrous heart valves, no fibrous tissue or scarring was observed. No differences were detected between KO and WT (see Supplementary S6 online).

Further histological differences were not obvious in our ultrastructural histological nor electron microscopy analysis (Fig. 3F), which do not reveal any significant difference between wt and dd-KO composition in closer muscle ultra-structure sarcomere assembly.

### Testing of c.899G > A variant in embryonic cardiac development in zfl using mRNA injection

**Fig. 4:**
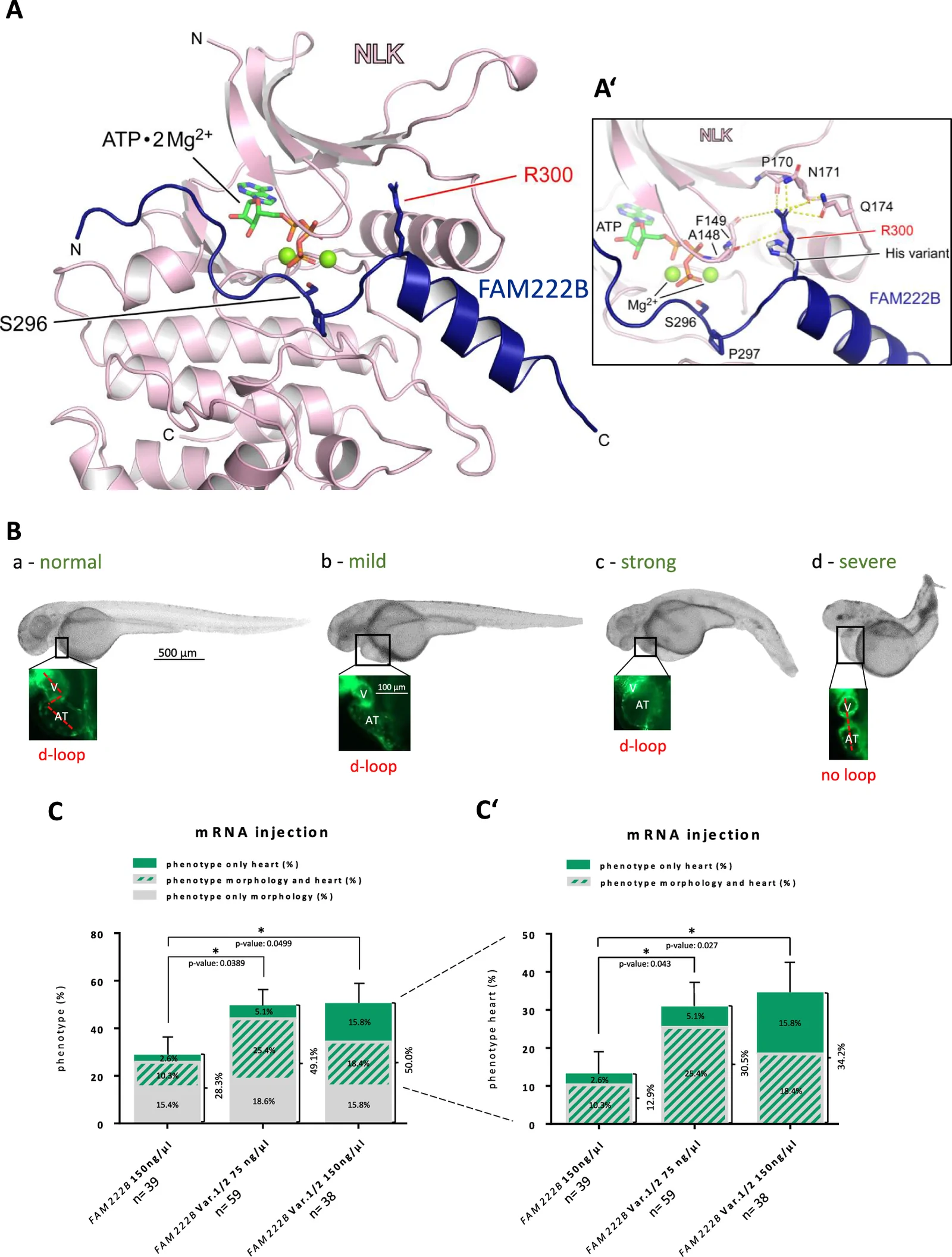
Modelling and functional effects of the *FAM222B* variant 1/2 rs753661690. (**A**) Structural model of human FAM222B (residues 286–314) bound to human NLK (residues 140–425). Arg300 (R300) of FAM222B is interacting with the β-sandwich structure of the kinase N-lobe, placing the preceding Ser296 (S296) residue in a position facing the bound nucleotide. Shown is the calculated model where the co-substrate ATP is complexed with two magnesium ions, with the γ-phosphate facing the hydroxyl group of Ser296 of the FAM222B sequence segment S_296_PISR_300_. (A’) Close up of catalytic centre showing hydrogen bonds between Arg300 and NLK as well as variant situation showing His300 lacking these. (**B**) Representative bright field overview and fluorescent heart images of poly(A) mRNA injected wt zfl. After mRNA injection, the 48 hpf old zfl were classified into four different severities of morphological and accompanying cardiac phenotypes: normal, mild (e.g. pericardial edema), strong (e.g. hypotrophic ventricle and concomitant dilated/ hypertrophic atrium) and severe (e.g. no heart looping and no grip). Scale bar: bright field: 500 µm, fluorescent cardiac region: 100 µm. (**C**) Bar chart classifies zfl (%) into three groups according to the specific developmental disorder: only morphological development (grey) affected, combined cardiac and morphological disorder (grey with green stripes) and only cardiac development (green) affected. N = 3, n = 38–59 in each group, in total. Overall phenotype: two-sided Chi^2^ test: 150 ng/µl wt against 75 ng/µl variant: p-value: 0.0389, *; 150 ng/µl wt against 150 ng/µl variant: p-value: 0.0499, *. Only morphologically changed zfl: two-sided Chi^2^ test: 150 ng/µl wt against 75 ng/µl variant: p-value: 0.677, ns; 150 ng/µl wt against 150 ng/µl variant: 0.961, ns. Combined phenotype, including a topical morphological and a cardiac phenotype together in one zfl: two-sided Chi^2^ test: 150 ng/µl wt against 75 ng/µl variant: p-value: 0.063, ns; 150 ng/µl wt against 150 ng/µl variant: p-value: 0.306, ns. Only cardiac altered zf: two-sided Fisher’s exact test: 150 ng/µl wt against 75 ng/µl variant: p-value: 1, ns; 150 ng/µl wt against 150 ng/µl variant: p-value: 0.056, ns. (C’) Bar chart accumulates all heart phenotypes (combined morphological + cardiac disorder in grey box with green stripes and only cardiac phenotype in green box) and is separately illustrated, meaning taken from (**C**), to underline differences in the amount of pathologically altered hearts in each group.

*FAM222B* encodes for an approximately 60 kDa nucleoplasmic and mitochondrial protein as annotated in the UniProt database. The protein does not contain any typical signal-peptide, transmembrane nor membrane-anchor regions^12^. Structure prediction by AlphaFold2 suggests an intrinsically unstructured protein with only five sparsely set helices within the 562 amino acids of the human variant. Through database research (STRING network) we could discover several potential interaction partners of FAM222B, including PDZD9, NLK, OR51D1, ZNF572, SSC4D, CEP152, OR2K2, ZNF789, TRMT10B and ZNF695 (listed according to scores). While these potential interaction partners were suggested from text mining, experimental evidence was only provided for NLK (STRING network: BioPlexExplorer, version BioPlex 3.0^20^; IntAct Molecular Interaction Database, version: 1.0.4^21^, both queries on 18th September 2025) which is also known to play an indirect role in cardiac development and function^22^. In a next step, we modelled protein complexes of FAM222B with the potential interaction partners, as many intrinsically unstructured proteins are known to adopt well-defined conformations only upon binding to their target proteins. Using AlphaFold3^23^, we calculated FAM222B complex structures with PDZD9, NLK, ZNF572, SSC4D and CEP152, always as heterodimers and heterotetramers (dimers of dimers). Following this approach, only the heterodimeric interaction of FAM222B with NLK revealed convergent results, with one helical element of FAM222B interacting with the N-lobe of NLK, suggesting the protein of interest as a substrate of the kinase. Notably, Arg300 of FAM222B mediates an intense interaction with the β-sandwich region of the N-lobe in all calculated models, positioning the phosphorylation-site Ser296 in the catalytic centre of the kinase (Fig. 4A). Moreover, it becomes apparent that the patient-derived histidine variant His300, rs753661690 cannot participate in the hydrogen-bonding network formed by arginine due to the significantly shorter side chain (Fig. 4A‘). This may comprise kinase recognition, resulting in the loss of phosphorylation of FAM222B. Of note, a recent study supported the interaction of FAM222B with NLK, suggesting that an N-terminal intrinsically disordered region in FAM222 family proteins interacts with the backside of the kinase, thus mediating the recruitment of the substrate^24^. Following these modelling results and considering the appearance of our found SNV p.300Arg > His in two unrelated families (Fig. 1A, cases 1&2), we focused on this mutation side in the zf model for further functional analysis.

Thus, we reconstructed this specific variant via mutagenesis of *FAM222B* wt DNA and investigated its effect on heart development performing poly(A) mRNA injection in *Tg(kdrl:EGFP)* wt zf embryos. For a clearer differentiation, we compared overexpression of human unmodified (wt) *FAM222B* mRNA to injection with the above-mentioned human missense variant rs753661690 SNV p.300Arg > His (var. 1/2) mRNA. We employed two different concentrations of mRNA for injections and compared the effects found on the zf ‘ morphological and cardiac phenotype to un-injected as well as embryos control-injected with phenol red and buffered water. After mRNA injection, the 48 hpf old zfl were classified into four different severities of morphological and cardiac phenotypes: normal, mild (e.g. pericardial edema), strong (e.g. hypotrophic ventricle and concomitant dilated/ hypertrophic atrium), and severe (e.g. no heart looping and no grip) (Fig. 4B). Statistical analysis was performed differentiating the distribution of all phenotypes (Fig. 4C).

Zf injected with *FAM222B* variant mRNA represent a higher percentage of phenotypes in total (75 ng/µl: 49.1%; 150 ng/µl: 50.0%) than zf injected with the overexpressed human wt *FAM222B* mRNA (150 ng/µl: 28.3%). This can also be found significant for both concentrations with a p-value of < 0.05* (Fig. 4C).

Whereas the number of only morphologically changed zf is approximately similar in each group: wt *FAM222B* 150 ng/µl: 15.4%; *FAM222B* rs753661690 (var. 1/2) 75 ng/µl: 18.6%; *FAM222B* rs753661690 (var. 1/2) 150 ng/µl: 15.8%, p-value: > 0.05, (Fig. 4C), the number of zfl with heart phenotypes in each group differs strongly:

Of note, the injection of *FAM222B* variant rs753661690 (var. 1/2) shows a cascading effect on the presentation of a cardiac phenotype. While the wt *FAM222B* 150 ng/µl group shows an isolated cardiac phenotype in only 2.6% of cases, this percentage increases in a dose-dependent manner to 5.1% in *FAM222B* rs753661690 (var. 1/2) 75 ng/µl and even 15.8% in *FAM222B* rs753661690 (var. 1/2) 150 ng/µl (p-value: = 0.0564) (Fig. 4C).

For a combined phenotype, including a morphological and a cardiac phenotype together in one zfl, it becomes apparent that an overexpression of wt human *FAM222B* mRNA led, with a phenotype percentage of 10.3%, to less developmental damage than the injection of the patients found variant rs753661690 with 25.4% in the lower and 18.4% in the higher concentration. This cannot be found significant for both concentrations (p-value: > 0.05) (Fig. 4C).

Notably, taken together all heart phenotypes (combined morphological + cardiac and only cardiac phenotypes), the wt *FAM222B* mRNA led with a total amount of 12.9% of cardiac altered zf to less heart developmental damage than the injection of the patients found variant rs753661690 with 30.5% in the lower and 34.2% in the higher concentration (Fig. 4C and separately highlighted in 4C’). This can also be found significant for both concentrations (p-value: < 0.05*).

From these results, and especially underlining the fact that heart phenotypes occur significantly more often after our variants mRNA injection, we conclude that variant 1/2, rs753661690, p.300Arg > His found in our affected patient might very well lead to a disturbed cardiac development.

## Discussion

Exome analysis of case-parent trios with cardiovascular laterality defects identified an identical de novo ultra-rare missense variant in the *FAM222B* gene. Survey of 2,109 single exomes of individuals with situs inversus totalis, heterotaxy, or isolated CHD identified five additional variants in *FAM222B* in five independent families. One of these five *FAM222B* variants is novel. Family EGAN00001389505 turned out to be a three-generation multiplex family with seven differently affected family members.

The frequencies of the missense variants reported in families 1, 2, 3 and 5 were significantly more common in the presented case cohort compared to the general population. The here reported nonsense indel variant identified in family 6, has not been described in gnomAD at all. In two families (family 4 and 7) the identified variant has the same frequency in our combined cohort here compared to the general population, arguing against its pathogenic effect (Fig. 1B).

As mentioned before, *FAM222B* has previously been considered a potential candidate gene for cerebral cavernous malformations^12^, but its embryonic function and protein role are still poorly understood and have not been explored in relation to CHD. Collectively, this data highlights *FAM222B* as a promising new candidate gene for cardiovascular laterality defects, leading us to investigate its role during zf embryonic development.

Our zf data revealed that the dd-KO of the two *fam222b* zf-paralogues, *fam222ba/bb*, has multiple effects on cardiac development. Both zf’ *fam*222 paralogues show a strong gene expression profile during early development in WISH analysis especially in the cardiac region (Fig. 2A). Furthermore, the gene knockout might well enhance the chance of a disturbed cardiac looping in the zfl (Fig. 2B,C). As shown in our data, cardiac looping was significantly more impaired in dd-KO zfl than in wt zfl (Fig. 2C). Other variables such as the zfl heart rate in bpm (Fig. 2E) do not seem to be affected by the knocked out *fam222ba/bb* genes. Although we focused on zfl heart rate, it is only one of several functional prognostic cardiac measures that might be affected.

We provide further evidence that *fam222ba/bb* not only affects zf in their larval stadium but that aberrant early cardiac development caused by a *fam222ba/bb* dd-KO might very well lead to disorders in adult age (≈ two years). This includes a significant enlargement of the dd-KO zf ventricle (Fig. 3B) which has previously been associated with heart failure^25^. The reduced cell density in dd-KO ventricular heart tissue, in the absence of intercellular gaps, suggests increased average cell size and is consistent with a hypertrophic heart phenotype. In parallel, the increased tissue area per unit mass indicates an expansion of tissue architecture relative to organismal weight. When integrating both parameters, dd- KO heart samples show an increased total cell number per mg despite reduced local cell density. Taken together, these findings indicate that the observed heart phenotype is likely driven by a combination of cellular hypertrophy and hyperplasia. In our studies, a decreased cell density under confluent conditions reflects hypertrophy whereas the increased total cell number per mg heart tissue supports hyperplasia. In many described human cases, a consequent enlargement of the atrium as also observed in our dd-KO adult fish hearts (Fig. 3C) results from ventricular dysfunction^26^. Other variables analysed in our study, such as the adult heart muscle ultra-structure and assembly do not seem to be affected by the knocked out *fam222ba/bb* genes. Disruption in sarcomere assembly or function often resulting in diverse cardiomyopathies^27^, could not have been detected in this study (Fig. 3F). Ventricular enlargement appears to result from hypertrophic growth, which was established through our comprehensive histological analyses, involving targeted nuclear staining and quantification (Fig. 3D,E).

Nonetheless, a limitation of this study arises from the fact that comprehensive analysis of cardiac functions requires analysis beyond zfl heart rate or comparison of heart morphology and histology, including additional parameters like, e.g., adult zf hearts’ ejection fraction, electrocardiogram and zf behaviour such as rapid breathing or exercise intolerance^25^. In order to percept multidimensional and more precise analysis of the zf heart, additional methods could be implemented. Greater measurement specificity could be achieved for example by including high-resolution echocardiography and electrocardiography^25^.

As mentioned above, we found an identical de novo variant (variant 1/2 rs753661690) of *FAM222B* in two independent families with left isomerism additional to complex CHD (Fig. 1A, cases 1&2). Via poly(A) mRNA injection of this missense variant within the human *FAM222B* gene, we showed that it causes significantly more cardiac harm to the injected zfl than purely the overexpressed wt *FAM222B* poly(A) mRNA (Fig. 4C,C‘). Based on this observation we conclude that it should not generally be the missing – in the case of KO – or excessive – in the case of overexpression – presence of FAM222B protein itself causing damage to the zf-heart, but rather the specific alteration in its amino acid sequence, in our case Arg > His at position p.300 heading to cardiac disorders. This conclusion was further supported by the structural modelling of FAM222B binding to NLK, which convincingly placed Ser296 of FAM222B at a site readily accessible for phosphorylation through the kinase, and proposed Arg300 to mediate key interactions with the kinase (Fig. 4A). Change of Arg300 to the shorter and uncharged histidine could impair the interaction with the kinase, possibly resulting in the loss of phosphorylation of FAM222B (Fig. 4A‘). However, it must be mentioned that most regions of FAM222B have a rather low confidence in the Alphafold prediction (UniProt), which is due to missing experimental reference structures within our protein of interest.

The various CHDs in the context of laterality defects in human individuals with heterozygous missense variants or deletion in *FAM222B* could only be recapitulated to a certain degree in the zf model presented here. It remains unclear whether each of our six identified variants individually gives rise to a distinct cardiovascular laterality defect phenotype in humans and zf, and exactly how. It is further questionable whether the cardiac phenotypes found in our mutant mRNA injected zf, e.g. hypotrophic ventricle and concomitant dilated/ hypertrophic atrium as well as no heart looping and no grip, could be precisely compared to the found human phenotypes e.g. VSD, ASD, AVSD and congenital bicuspid aortic valve. Due to fundamental differences in cardiac anatomy between zf and humans, such as the presence of only one atrium and ventricle (two-chambered heart) in zf in comparison to the human four-chambered heart, certain structural defects observed in our patients like the here described VSD or ASD cannot be reconstructed properly in this animal model. A more anatomically comparable model, such as mouse, which possesses two atria and two ventricles, could well provide more meaningful and well-founded evidence to this question. The here identified additional expression pattern of *fam222ba/bb* in the cloaca, pineal gland, and the cranial head region of the zfl (see Supplementary S3 online) may offer important insights into our candidate gene’s role across different other tissues and diseases and could therefore well hold relevance for further studies. Collectively, the here presented human genetic and zf data strongly support an important role for *FAM222B* in the pathogenesis of cardiac diseases and potentially in early cardiac axis development as well.

## Materials and methods

### Exome analysis of case-parent trios and single exomes in individuals with cardiovascular laterality defects

DNA isolation and exome sequencing of affected case-parent trios (HET15 and HET17) was carried out consenting to the IRB ethical approval and authorized by the local ethics committee of the medical faculty of the University of Bonn (Lfd Nr. 141/15), as previously published for other laterality defects associated disease genes by Breuer et al. in 2022^11^. Since family members of the index case might present with clinically unapparent features of the laterality defect spectrum, all family members included received a thorough clinical exam and ultrasound studies by two paediatric cardiologists in order to assess the affection status of all study participants^11^. Furthermore, all patients included were assessed for extracardiac manifestations. In the patients described here, no extracardiac manifestations were found. Single exome data was retrieved from an earlier study by Sifrim et al. 2016^28^. Filtering of exome data was performed as published earlier by Breuer et al. in 2022^11^.

### Zebrafish husbandry and Embryo preparation

Under provided husbandry §11-license of the Zebrafish Core Facility in Bonn, zf are maintained according to national law and to recommendations by Westerfield^29^. The adult zf were kept in a 28°C temperature 3.5-L tank in a water-recirculating rack-system on an adequate 14-h light and 10-h dark cycle. Zfl of wt AB/TL, *Tg(kdrl:eGFP;gata)*; s844Tg (ZDB-ALT-051214–6) and *Tg(kdrl:eGFP;gata ds:red) fam222bb* hu10539; *fam222ba* hu10755; s843Tg (ZDB-FISH-161130–1, s843Tg) TALEN derived strain were obtained by natural spawning and raised in an incubator at 28°C in 30% Danieau’s buffer longest until independent ingestion on day five post fertilisation. For imaging and WISH-analysis performed at ≤ 5 days post fertilization pigmentation was suppressed by adding 0.003% phenylthiourea (PTU) to the Danieau solution.

### *Fam222ba/bb* dd-KO

TALEN- derived *Tg(kdrl:eGFP;gata ds:red) fam222bb* hu10539; *fam222ba* hu10755; s843Tg (ZDB-FISH-161130–1) strain obtained from B.C. Kirchmaier, Goethe University Frankfurt, and firstly described by S. Spiegler, University of Greifswald, were spawned for our experiments. TALE nuclease injection and non-homologous end-joining led to a 10-base pair deletion in the *fam222ba* gene *(fam222ba hu10755*) 5 ‘*-*TTGTCCATGG-3 ‘ and in the *fam222bb* gene (*fam222bb hu10573*) 5 ‘-CGGACGGGCC-3 ‘, respectively, resulting in a frameshift with a premature stop in the protein coding sequence. These deletions were confirmed by resequencing during the time of experiments.

### Sequencing and Genotyping of *fam222ba/bb* dd-KO line

For sequencing and genotyping of the received *Tg(kdrl:eGFP;gata ds:red) fam222bb* hu10539; *fam222ba* hu10755; s843Tg (ZDB-FISH-161130–1) TALEN derived strain, PCR was performed on skin-swabbing^30^ derived genomic DNA with HOT FIREPol® Blend Master Mix Ready to Load (Solis Biodyne, Cat. Num. 04–25-02,025). For sequencing, the PCR-product was sent to Eurofins Genomics, Bonn. Primer sequences are listed in supplemental material, primer.

### Whole-mount zf in situ hybridization

For preparation of sense and antisense probes of *fam222ba*, *fam222bb*, *fam222aa* and *myl7*, cDNA plasmids were generated by RT-PCR amplification and then cloned into the SK(-) pBluescript® vector. As a PCR template, cDNA of 56 hpf old wt AB/TL zfl was used. For probe preparation of *fam222ba*, *fam222bb*, *fam222aa* and *myl7* digoxigenin-labelling was performed using the DIG RNA Labeling Kit (Cat. No. 11 175 025 910) from Roche. Double staining with an additional fluorescein-labelled *adprhl1* hard specific probe was performed using the same Kit but instead of DIG RNA Labeling Mix (Cat. No. 11 277 073 910) Fluorescein RNA Labeling Mix (Cat. No. 11 685 619 910) from Roche was used. High resolution in situ hybridization on whole-mount zf embryos was established according to Thisse, C.& Thisse, B.^31^. Double-staining was performed according to Moens, C.^32^ https://research.fredhutch.org/content/dam/stripe/moens/other/RNA_in_situ_protocol.pdf.

Prior to experimental procedure, the zfl for WISH analysis were treated with 1-phenyl 2-thiourea (PTU) Danieau solution in order to prevent natural pigmentation. Images were taken using the Nikon Eclipse Ni-U Microscope with DS-Fi2 Camera and the NIS-Elements BR 5.20.00 64-bit software. For general expression analysis of the zf ‘ *fam222b* paralogues and *fam222aa*, zfl were positioned laterally (Fig. 2A). For imaging analysis of *myl7* cardiac looping (Fig. 2B) the zfl tails were cut off, zfl were placed on the stump in a drop of glycerol and imaged from frontal towards cranial.

### Heart dissection of adult zf

For heart preparation, approximately two-year-old zf were euthanized by hypothermic ice shock and fixed in a lateral position left side up on a dissection Styrofoam plate. The skin was cut from the pelvic fin along the belly of the zf to the operculum. After a 90-degree cut towards the upper edge of the zf eye, the skin and subcutaneous fat was unfolded to expose the heart. The dissected heart was then washed in PBS-buffer and imaged using the Zeiss Stemi 508 ZOOM Stereomicroscope. All area measurements were performed using Fiji ImageJ version 1.53c, Java 1.8.0_172, win64-bit (National Institutes of Health, USA, Wayne Rasband) measuring software.

### Haematoxylin–eosin (HE) staining

The dissected hearts were fixed in 4% paraformaldehyde in PBS-buffer; pH 7.4 and then dehydrated. After this, they were embedded in paraffin*,* cut in ~ 12 µm sections and stained with HE. For HE-staining an intern protocol was followed.

### Masson’s trichrome stain

The dissected hearts were fixed in 4% paraformaldehyde in PBS-buffer; pH 7.4 and then dehydrated. After this, they were embedded in paraffin*,* cut in ~ 12 µm sections and stained using Masson’s trichrome stain. Staining was performed according to a modified in-house protocol for zf.

### Transmission electron microscopy (TEM)

For TEM of the adult zf heart, samples underwent fixation with a solution based on cacodylate buffer containing 1.5% Glutaraldehyde, 1% Paraformaldehyde and 3% Sucrose. The samples were then rinsed in phosphate buffer and subsequently post-fixed for 1 h at room temperature in aqueous 2% osmium tetroxide in phosphate buffer. A graded ethanol series for dehydration was followed by a transition to propylene oxide and an embedment in Spurr epoxid resin. Slices of 1 µm and 50 nm thickness were cut with a diamond knife on a Reichert-Jung Ultracut E Microtome 70 17 74 and mounted on formvar coated single slot grids. Contrasting with aqueous solution of 0.5% uranyl acetate and 3% lead citrate was realised prior to examination in transmission electron microscope (Jeol JEM-1400 Plus).

### Structure modelling

Complex structures of human FAM222B (UniProt accession code Q8WU58; 562 aa) bound to various proposed interaction partners were modelled with AlphaFold3^23^, either as heterodimers or hetero-tetramers (dimer of dimers) using full-length sequences. Best results were achieved for the 1:1 stoichiometric complex with the human Ser/Thr-kinase NLK (UniProt Q9UBE8), where R300 of FAM222B was proposed to mediate intensive interactions with NLK. Addition of ATP and two magnesium ions led to the calculation of a kinase active model, where Ser296 of FAM222B is facing the catalytic centre. Protein graphics were generated using the PyMOL Molecular Graphics System (Version 2.5.5 Schrödinger, LLC).

### Mutagenesis and mRNA Synthesis of human *FAM222B*

For overexpression and mutagenesis experiments, a cDNA IMAGE clone 4,040,385 (Art. Num. IRAUp969A0655D) of human *FAM222B* cloned into pOTB7 Vector was obtained from Source BioScience.

Mutagenesis of human variants into wt *FAM222B* was performed using an Agilent Technologies QuikChange Lightning Site-Directed Mutagenesis Kit (Cat. Num. 210,519–5; for Variant 1/2) and a New England Biolabs Q5® Site-Directed Mutagenesis Kit (Cat. Num. E0552S; for Variants 3–7). Kit associated protocols were used.

After linearization of respective cDNA plasmid, mRNA transcription and poly(A) tailing were performed using the mMessage mMACHINE® Kit (SP6; Cat. Num. AM1340) and the Poly(A) Tailing Kit (Cat. Num. AM1350) by Invitrogen (Thermofisher). The kit-attached protocols were followed.

### Microinjections of mRNA

In one to two-cell *Tg(kdrl:eGFP;gata)* transgenic wt embryos, overexpression of human *FAM222B* mRNA was performed by injecting 0.3 ng (2 nl at 150 ng/ul) of the wt mRNA into the embryos ‘ yolk sac. We prioritized the *FAM222B* variant 1/2 (rs753661690, c.899G > A, p.Arg300His) because of its independent appearance in two non-related families and its characteristics in protein structure modelling. Poly(A) mRNA of rs753661690 was injected in two different concentrations: 75 ng/µl (0.15 ng mRNA) and 150 ng/µl (0.3 ng mRNA). As a control, un-injected and embryos sham injected with a phenol red, water mix were compared. For pressure injection through mechanical-manipulator positioned glass-capillaries the “Milli-Pulse Pressure Injector, Model MPPI-3” (Applied Scientific Instrumentation, Inc. 29,391 W. Enid Rd. Eugene, OR 97,402–9533, United States) was used.

### In vivo fluorescent zfl imaging

Phenotype evaluation after mRNA injection was performed at 48 hpf using the Zeiss AXIO Zoom.V16 microscope equipped with ZEN 2.6 pro software. For statistical analysis, we performed a dead zf quantification at 24 and 48 hpf as well as a phenotype observation on remaining zfl at 48 hpf. The cardiovascular system was analysed using the green fluorescence reporter-signal of the *Tg(kdrl:eGFP;gata)* transgenic strain.

### Statistical analysis

Statistical analysis was performed using GraphPad Prism version 6.01 for Windows. The alpha level was set to α = 0.05. Differences with a p-value of < 0.05* are mentioned as statistically significant and p-value < 0.01** as very significant within the main text. Data distribution, statistical test forms and exact p-values of each test are given in the according figure legend. For binary, nominally scaled data two-sided Chi^2^-test without Yates’ correction (expected frequency > 5) or two-sided Fisher’s-exact-test (expected frequencies < 5) were used, for ratio scaled data unpaired two-tailed t-test was used. Normality of ratio scaled data was verified for all groups using D’Agostino-Pearson (n > 25) or Shapiro–Wilk-test (n < 25). Nominally scaled data error bars were created according to individual SEₚ.

### Regulatory and guideline compliance statement

We do confirm that all methods were performed in accordance with the relevant guidelines and national regulations.

## Supplementary Information

Supplementary Information.

## Data Availability

All data relevant to this study and supporting the conclusions of this article are included in the article or attached in the supplementary information. Raw whole-genome sequencing data are not publicly available due to restrictions imposed by the participants’ informed consent and ethical approval. These data are available from the corresponding author upon reasonable request and will be shared with qualified researchers via secure data transfer platform in accordance with the applicable ethical and consent requirements.
