# Supplementary material for: Molecular characterization of FAM222B as a novel disease gene for dominant cardiovascular laterality defects
Source: Sci Rep. 2026 Aug 22;16:26359. doi: 10.1038/s41598-026-64853-6 (PMC13499801; doi:10.1038/s41598-026-64853-6)
Supplement: Supplementary file 1 [file 41598_2026_64853_MOESM1_ESM.pdf]

supplementary

2025/26 scientific reports

congenital heart defects: diagnosis and treatment

Nina Reitz

Supplementary **S1**

Var.	Fam222ba	Fam222bb	Fam222aa
1/2	yes	yes	no
3	no	yes	no
4	yes	yes	yes
5	no	no	no
6	partial-R**PQ**T**L**CH	partial-**H**Q**Q**Q**L**SA	no
7	no	no	no

S1: Comparative analysis of conservation of the observed human variants in zf on amino acid level within the zf Fam222b homologues and Fam222a. Fam222bb presents the highest concordance with three out of six conserved human variants in zf. Conserved amino acids are indicated in bold letters.

Supplementary **S2**

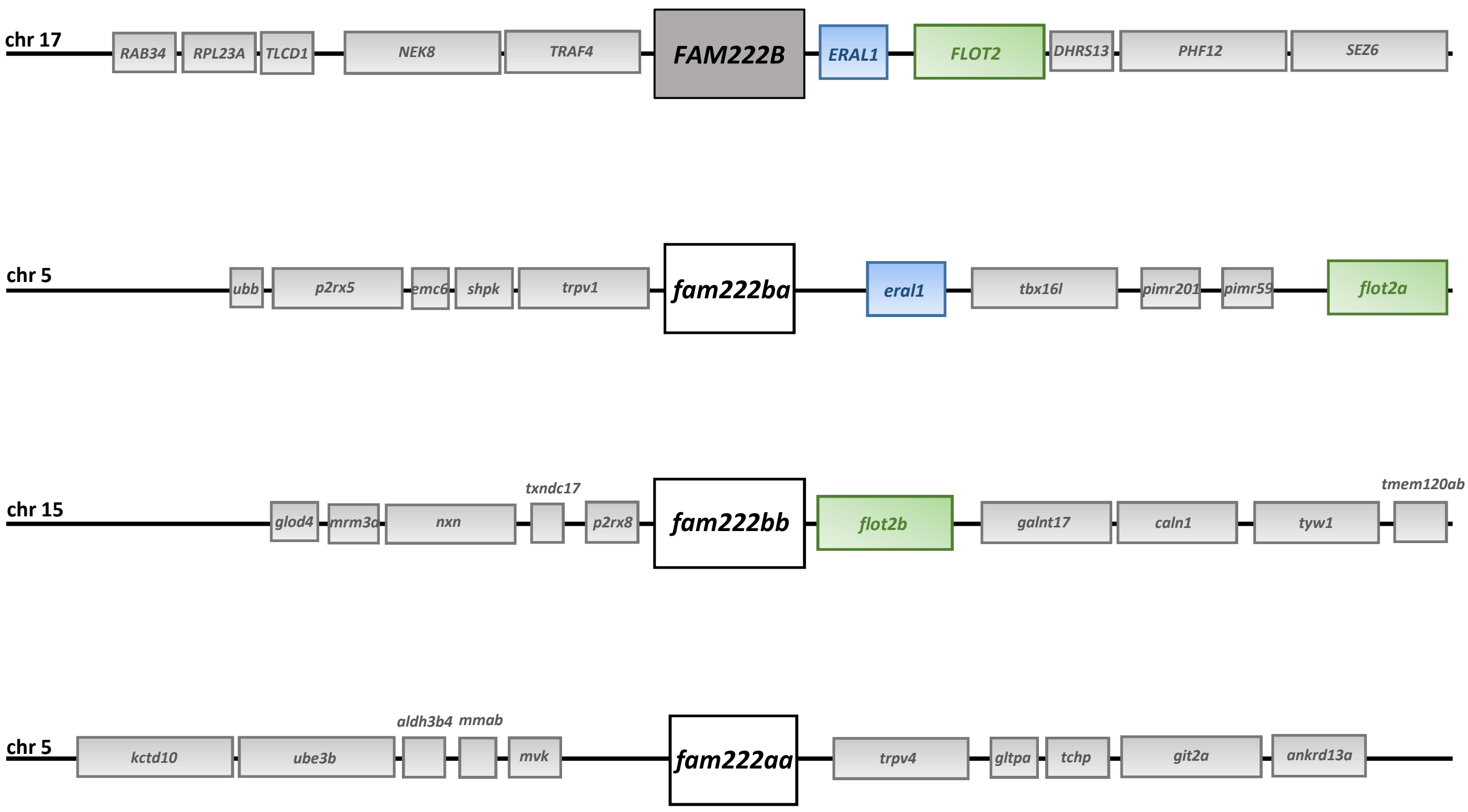

S2: Comparison of up- and downstream-localised neighbouring genes between human *FAM222B*, the zf paralogues *fam222ba/bb* and zf *fam222aa.* Concordant genes are marked by the same colour, which shows the highest conservation of *FAM222B* in this case with *fam222ba followed by fam222bb* and no common gene neighbours with zf *fam222aa.*

Supplementary **S3**

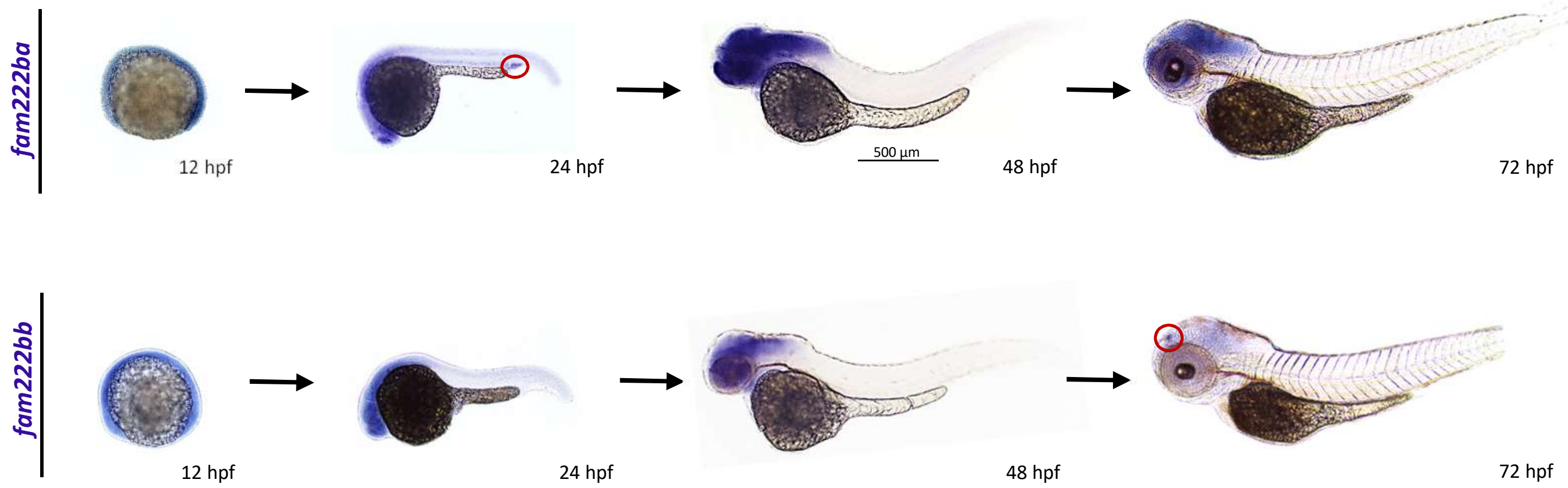

S3: Timeline of whole-mount in situ hybridization (WISH) analysis of *fam222ba* (top) and *fam222bb* (bottom) expression at various time-points from 12 hpf until 48 hpf. The zf head and cardiac region are strongest stained at 48 hpf. At 24 hpf *fam222ba* is also expressed in the cloaca, at 72 hpf *fam222bb* is expressed in the pineal gland (marked with red circles). Scale bar: 500 µm.

Supplementary **S4**

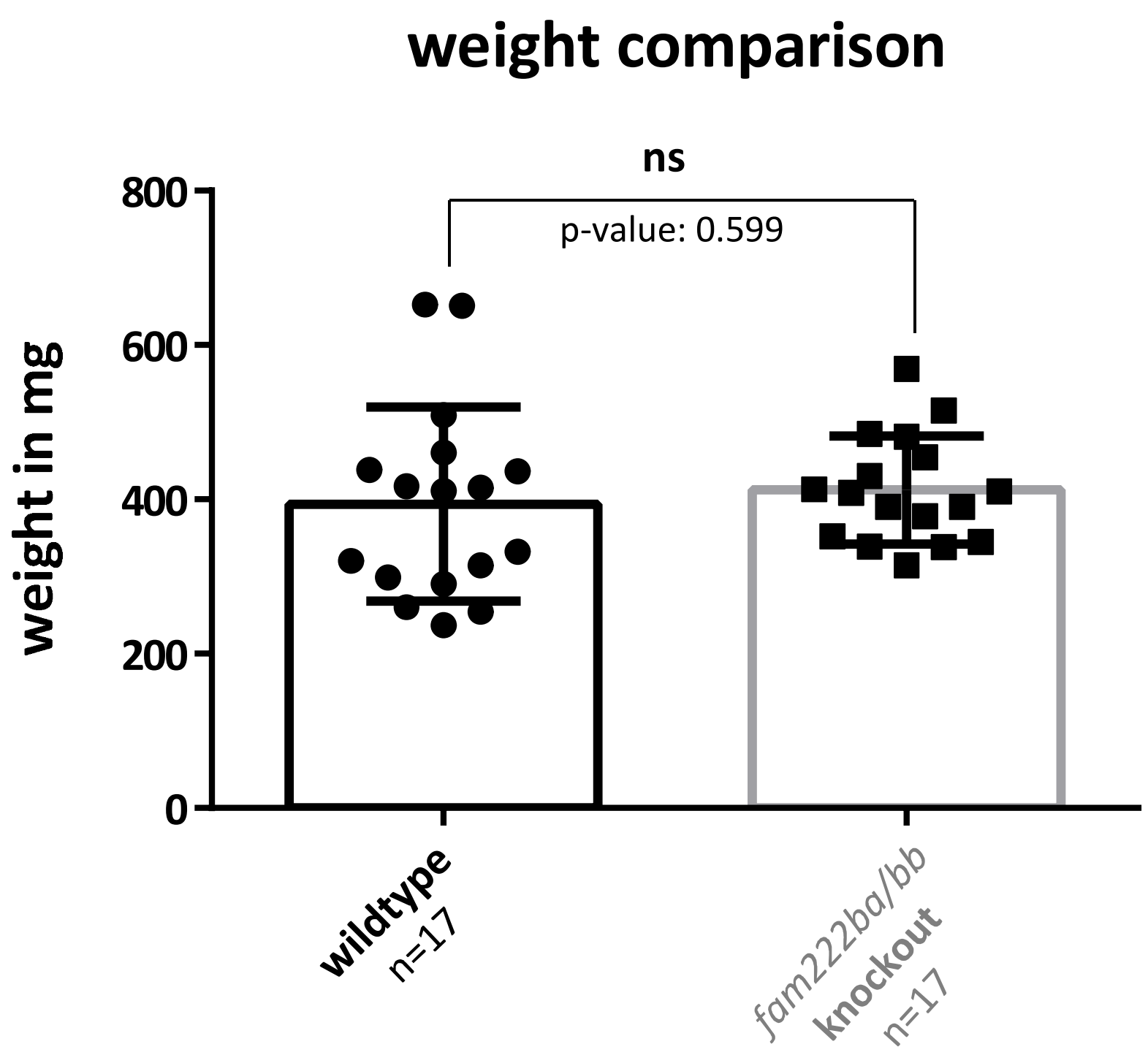

S4: Weight comparison of adult wt and *fam222ba/bb* dd-KO zf for 2D- area measuring (N= 4; n= 17 in each group, in total). No significant difference between both groups. Median (mg): wt: 411, dd-KO: 408. Mean (mg): wt: 393.8, dd-KO: 412.4. Unpaired two-tailed t-test: p-value: 0.599, ns= not significant.

Supplementary **S5**

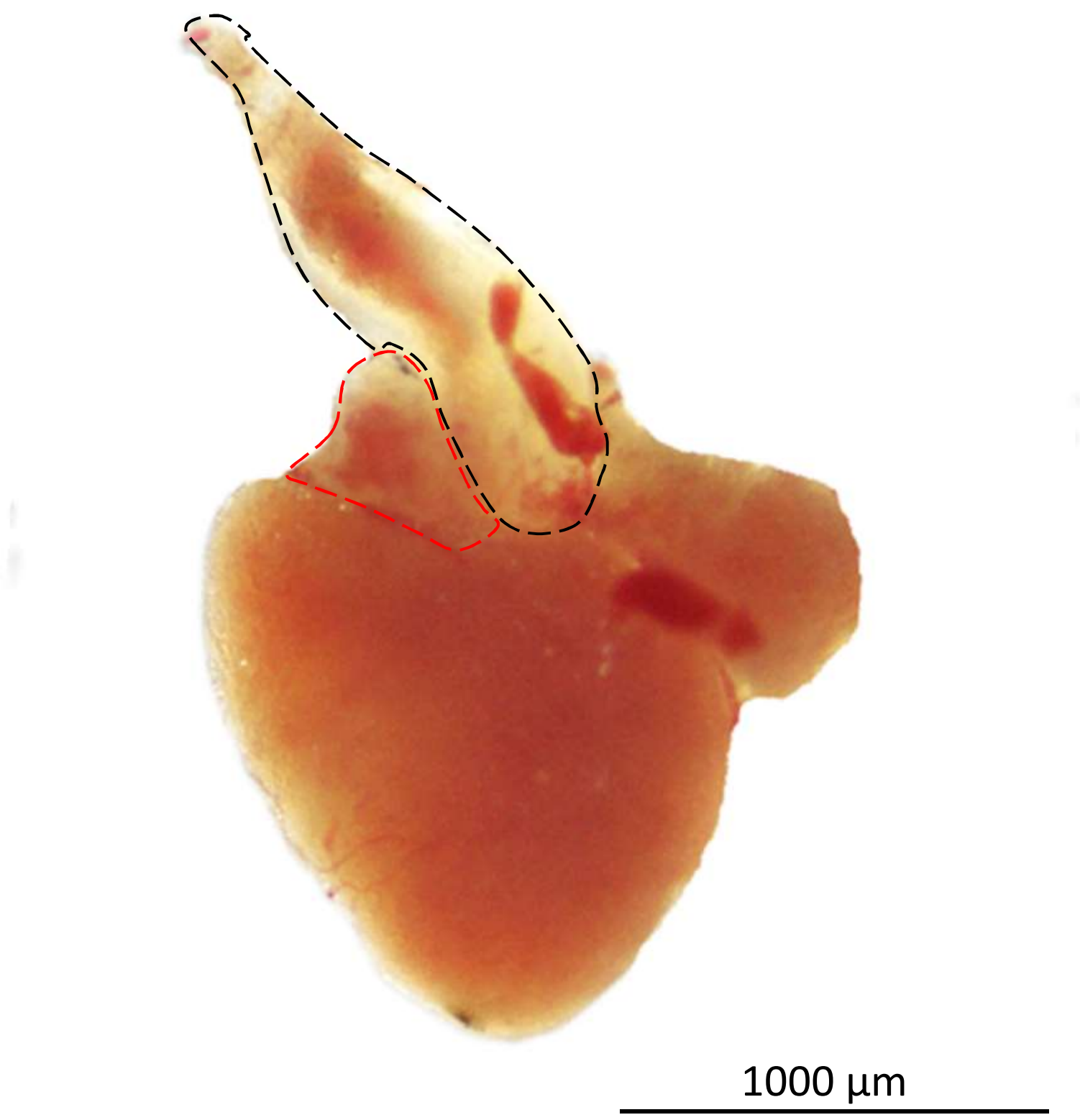

S5: Detailed examination of adult *fam222ba/bb* dd-KO zf heart with double outlet BA. This particular case was observed in only 1/16 knockout hearts. Dashed lines symbolize the way of measurement: primary BA in black, secondary BA in red. Scale bar: 1000 µm.

Supplementary **S6**

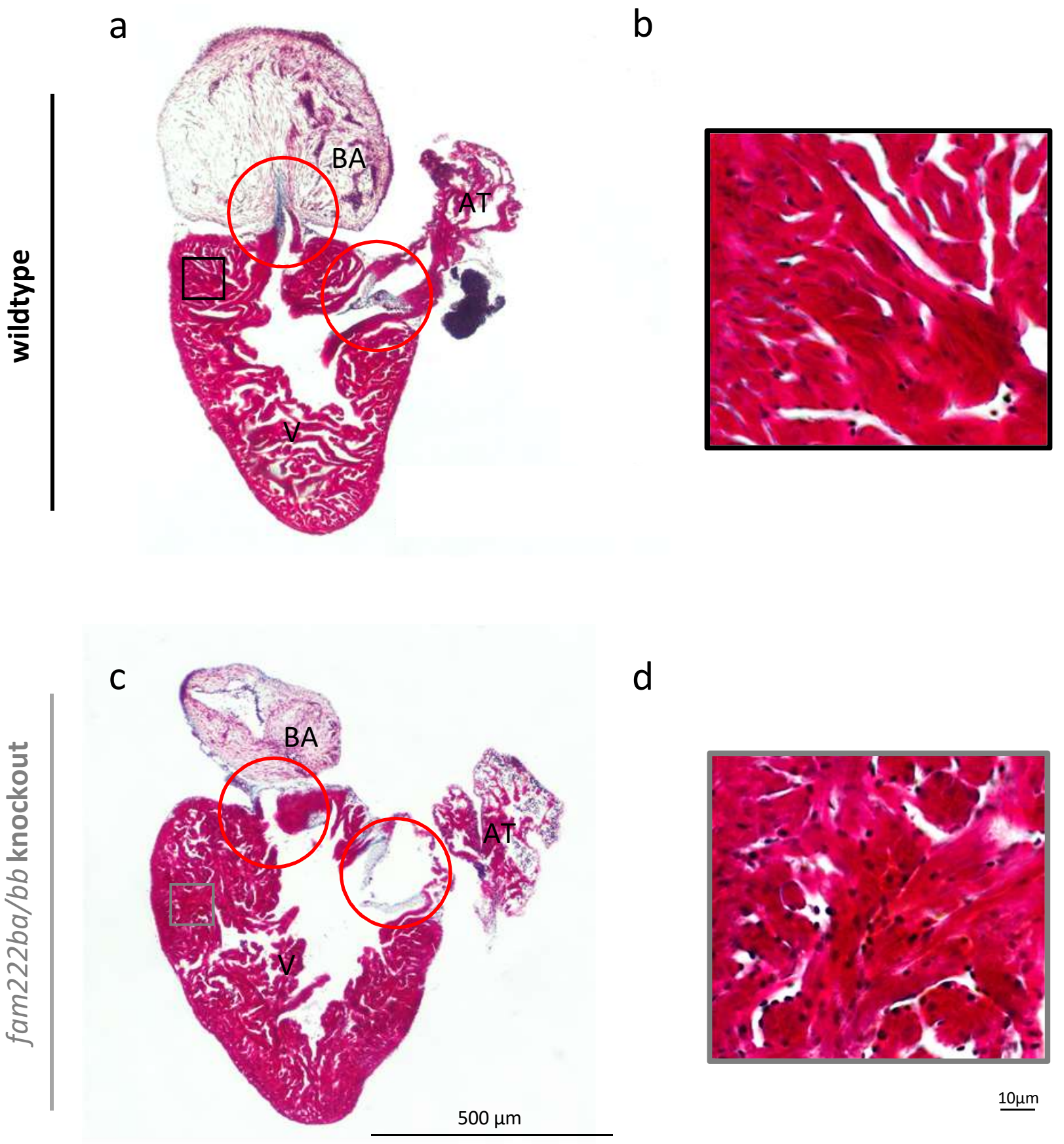

S6: Masson's Trichrome Stain of adult wt (a,b) and dd-KO (c,d) zf hearts (N= 3; n= 8 in each group, in total). Boxes in the overview panels (a, c) indicate regions shown as higher-resolution magnified panels (b,d). Scale bar: overview: 500 µm, zoom: 10 µm. Abbreviations: AT= atrium, BA= bulbus arteriosus, V= ventricle. Red circles around light green stained heart valves. With the exception of the physiological fibrous heart valves, no fibrous tissue or scarring was observed. No differences were detected between KO and WT.

supplemental material - primer

primer name	usage	sequence	f/ r
fam222b hum 1F	sequencing	GTAAAACGACGGCCAGT	f
fam222b hum 14R	sequencing	CAGGAAACAGCTATGAC	r
c.899G>A mut F	mutagenesis agilent	CCCAGCCCCATTAGTCACAGTCTGCTCATCAAT	f
c.899G>A mut R	mutagenesis agilent	ATTGATGAGCAGACTGTGACTAATGGGGCTGGG	r
rs1247152952	mutagenesis Q5	TGCCATTGTCGAAGTGCCAGC	f
rs1247152952	mutagenesis Q5	AGCAGGCCTGCCTTGGTG	r
Variant 5 C>T	mutagenesis Q5	GCTACTGTGGTACCCAGCACTTTAG	f
Variant 5 C>T	mutagenesis Q5	ATAGGGTGCCACTGGGGG	r
rs768625209	mutagenesis Q5	CAGCATGCACTGACCCTGGCCCATGC	f
rs768625209	mutagenesis Q5	CAGGGCCTGCTGGCGGGC	r
rs758359508	mutagenesis Q5	GCTAGTGGGATGCCAGCCCCT	f
rs758359508	mutagenesis Q5	CTCGCTGCACATCTGCTGTAG	r
deletion F Q5	mutagenesis Q5	GCCTCAGGGCCTGGGCCA	f
deletion R Q5	mutagenesis Q5	TGGGACAGTGCCTGGGGTG	r
fam222ba_1F	cDNA cloning & sequencing	tcatGGTACCATGCTGGCCTGTCTGCCA	f
fam222ba_1R	cDNA cloning & sequencing	tcatGGATCCTTATCTGTACCCTGGGTGCTGG	r
Fwd Primer pblue fam222bb	cDNA cloning	tcatGGTACCACAATCGCACGTCTCATCC	f
fam222bb 1R control	cDNA cloning	tcatACATGTAGCCATAGCTCATCTGTACC	r
fwd 3' probe in situ ba	in situ Sonde ba 3'	tcatGGTACCGTCGGTGCATGCAGGAAAC	f
rev 3' probe in situ ba	in situ Sonde ba 3'	tcatGCGGCCGCTTATCTGTACCCTGGGTGCTGG	r
fwd 3' probe3 bb	in situ Sonde bb 3'	tcatGGTACCATGGGTGGCGACTTCCAGA	f
rev 3' probe3 bb	in situ Sonde bb 3'	tcatGCGGCCGCTCCCAGTATCAGTTTGCACTTTC	r
Fwd modified fam222a	in situ probe synthesis	TCATGGTACCTGCACCCCAGCACAACCCAG	f
Rev fam222a	in situ probe synthesis	TCATGCGGCCGCCTGGTATCACCGTCCCTGAA	r
re-seq_F adprhl1	sequencing	CTGATCTCCGGCTGTCTGTTC	f
re-seq_R adprhl1	sequencing	GGGGATCCAGACATGATAAG	r
fwd2 myl7	in situ myl7 probe synthesis	tcatGGTACCCGAACAGAAACACTGCAGAC	f
fwd3 myl7	in situ myl7 probe synthesis	tcatGGTACCGCAAAGCAGACAGTGAACA	f
rev1 myl7	in situ myl7 probe synthesis	tcatGCGGCCGCGGTTTGAGATGATGCTCTACTC	r
M13-fwd	sequencing	TGTAAAACGACGGCCAGT	f
M13-rev	sequencing	CAGGAAACAGCTATGAC	r

supplemental material - IDs

	Ensembl ID	Zfin ID	UniProt ID
**FAM222B**	ENST00000581407.6 -208	/	Q8WU58
**fam222ba**	ENSDART00000145247.2 -201	ZDB-GENE-060526-355	A0A8M9PK94
**fam222bb**	ENSDART00000154504.2 -201	ZDB-GENE-110419-4	E7F6C1
**fam222aa**	ENSDART00000131924.2 -201	ZDB-GENE-050309-42	A0A8M2BCF5
